# Canthaxanthin Retinopathy with Visual Loss: A Case Report and Review

**DOI:** 10.1155/2013/140901

**Published:** 2013-10-30

**Authors:** Robert A. Beaulieu, Ronald E. Warwar, Bruce M. Buerk

**Affiliations:** ^1^Department of Surgery, Wright State University Boonshoft School of Medicine, 3640 Colonel Glenn Highway, Dayton, OH 45435, USA; ^2^Division of Ophthalmology, Wright State University Boonshoft School of Medicine, 3640 Colonel Glenn Highway, Dayton, OH 45435, USA

## Abstract

Canthaxanthin is a naturally occurring chemical, which is most commonly utilized as a colorant for food and dyes or a skin bronzing agent. Its most prevalent impact on human health is canthaxanthin retinopathy, which appears as birefringent, yellow to red crystals surrounding the macula. This occurs with increasing, dose-dependent exposure. Generally, patients remain asymptomatic and findings may only be evident on funduscopic examination. Cessation of canthaxanthin ingestion appears to reverse the retinopathy, but the time until crystal disappearance is variable. Despite a usually favorable outcome, long-standing visual changes may occur. We report a case of an 84-year-old woman with significant visual loss secondary to canthaxanthin retinopathy that ultimately improved upon cessation of the drug.

## 1. Introduction

Canthaxanthin is a naturally occurring carotenoid pigment which is synthesized by microorganisms and plants. It can be found in fruits, vegetables, and fish and primarily occurs in human tissue as a result of dietary ingestion. Like other carotenoids, it is fat-soluble and intensely colored. Canthaxanthin carries a red to orange hue and is used as an agent for coloring foods, dyes, and for skin bronzing. Experimental use has been successful for photoprotection for erythropoietic protoporphyria and cosmetic improvement in vitiligo [1]. The bronzing effect is achieved through carotenoid deposition in the dermis and subcutaneous tissue. Past reports have concluded that this chemical does not carry genotoxic, reproductive, or carcinogenic risks and does not have allergic potential as an oral medication and that acceptable daily intake is 0.03 mg/kg/day [2]. However, there have been reports of adverse impacts on human health, mainly canthaxanthin retinopathy, which manifests as birefringent, yellow to red crystals in the macula [3, 4]. In addition, a case of aplastic anemia has been reported in association with canthaxanthin ingestion [5]. We present a case of an elderly woman with visual loss from canthaxanthin retinopathy following years of use of the drug.

## 2. Case Report

**Figure 1:**
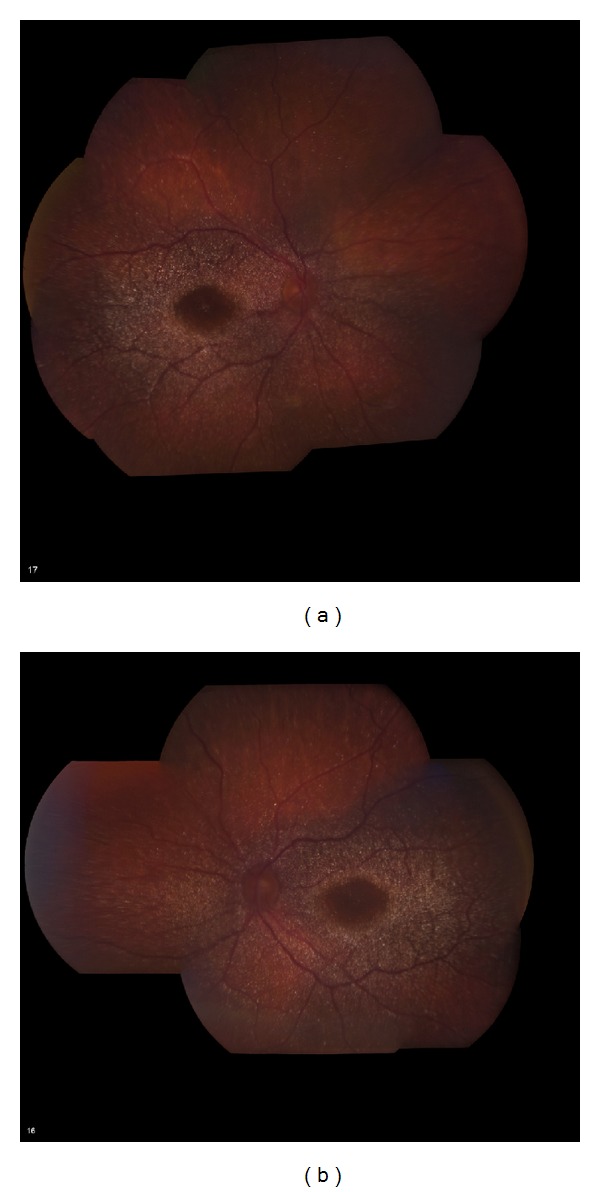
Fundus photographs of an 84-year-old woman showing diffuse canthaxanthin crystal deposition. (a) Right eye. (b) Left eye.

**Figure 2:**
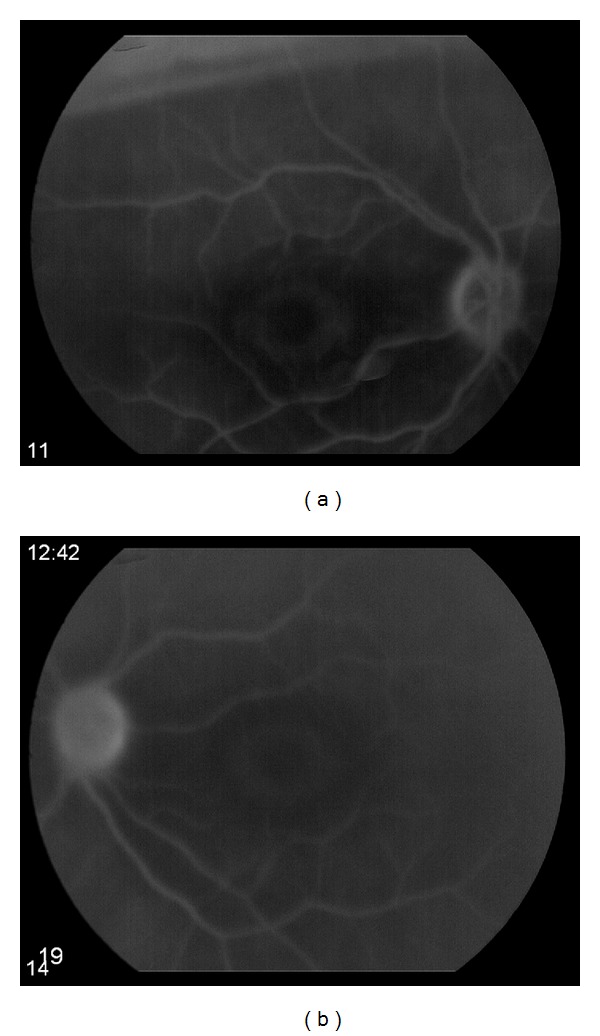
Late phase fluorescein angiogram of an 84-year-old woman with canthaxanthin retinopathy showing bull's eye pattern of central hypofluorescence. (a) Right eye. (b) Left eye.

**Figure 3:**
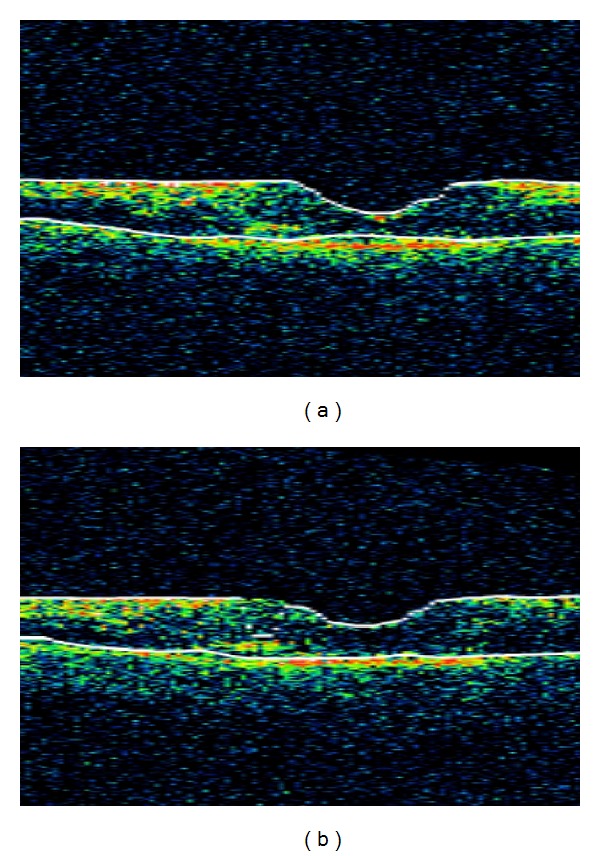
Optical coherence tomography of an 84-year-old woman with canthaxanthin retinopathy showing a thin retina with no edema, crystals, or shadowing artifacts. (a) Right eye. (b) Left eye.

An 84-year-old woman presented with a two-month history of worsening vision in both eyes. She had a 10-year history of canthaxanthin use for tanning, at variable dosages. Estimated total dosage is greater than 100 g. She had undergone bilateral cataract extractions in the past and had no other known ocular diseases. Visual acuity was 20/200 OD and 20/300 OS. Funduscopic examination demonstrated bilateral macular pigmentary changes and gold crystalline deposits (Figure 1). Fluorescein angiography demonstrated good perfusion of the retina and choriocapillaris. There was central hypofluorescence noted in the macula from blocking defects with a discernible bull's eye pattern noted in the late phases of the study OU. There was no leakage OU (Figure 2). Optical coherence tomography (OCT) showed a thin retina. No macular edema, crystals, or shadowing artifact was noted (Figure 3). The patient was instructed to discontinue the canthaxanthin. One month later, visual acuity improved to 20/70 OD and 20/60 OS. Eighteen months later, visual acuity stabilized at 20/40 in both eyes, despite the funduscopic persistence of crystals.

## 3. Discussion

Canthaxanthin and other carotenoids locate in biological lipid membranes and based on their orientation and location can influence membrane properties such as permeability and fluidity. They are powerful antioxidants due to their radical scavenging and singlet oxygen-quenching properties [6]. However, in vitro studies have actually demonstrated prooxidant effects of carotenoids at higher concentrations [6]. In the setting of canthaxanthin retinopathy, it is thought that damage occurs at the level of the macular vascular system around areas of canthaxanthin-lipoprotein complex deposits, which comprise the visible crystals [7]. It is proposed that vascular dysfunction occurs due to aggregation of these complexes in vessel lipid layers, which modify and disrupt lipid membrane properties [7].

Incidence and prevalence are difficult to predict due to the generally asymptomatic course of canthaxanthin retinopathy. Some reports state an incidence between 12 and 14% [8, 9]. Harnois et al. [9] noted that crystal appearance follows a dose-dependent correlation, seen with 50% of patients ingesting a total dose of 37 g and with 100% ingesting greater than 60 g.

Funduscopic examination generally reveals highly reflective, tiny (30 *μ*m) crystals accumulating in the perifoveal area. Daicker et al. [10] reported that, when examined posthumously, light microscopy revealed birefringent crystals in the inner layers of the entire retina, and analysis with ultrahigh resolution OCT has shown that canthaxanthin retinopathy crystals are found in the outer plexiform layer [11]. While the vast majority of patients with canthaxanthin retinopathy remain asymptomatic, visual field defects, decreased visual acuity, abnormal electroretinogram testing, and low static luminance threshold may be present [12, 13]. Decreased static perimetry testing in patients with canthaxanthin retinopathy compared to control group was described by Harnois et al. [13], with a positive correlation with total drug dosage. On long-term followup, static perimetry testing returned to normal following discontinuation of the canthaxanthin. While often normal, fluorescein angiography may show a perifoveal ring of blocked fluorescence corresponding to areas of crystal deposition [14–16]. 

The present case, to our knowledge, represents the oldest reported patient with canthaxanthin retinopathy. Also relatively unique to this case is the severe decrease in visual acuity. Such a complication is likely related to the large total ingested dose compounded by the patient's age, similar to other drug-related retinopathies such as that associated with hydroxychloroquine [17]. 

Differential diagnosis for canthaxanthin retinopathy includes other crystalline disorders such as tamoxifen, methoxyflurane, and talc retinopathy. Primary ocular disorders such as Bietti's crystalline retinopathy, calcified macular drusen, and idiopathic parafoveal telangiectasis are other possibilities. Crystals on the inner retinal surface in the setting of longstanding retinal detachment have also been reported, although the pathophysiology of this finding has not been fully elucidated [15]. Finally, systemic diseases such as oxalosis, cystinosis, hyperornithinemia, and Sjögren-Larsson syndrome should also be considered [18]. 

Treatment for canthaxanthin retinopathy is immediate discontinuation of the drug as soon as crystals are identified, even if the patient is asymptomatic. Prognosis is very good with complete recovery occurring in the vast majority of patients. Hueber et al. [16] followed five patients for 16–24 years and no long-term adverse effects were found, and fluorescein angiography results were normal, although complete resolution of golden particle appearance took up to 20 years. 

In summary, canthaxanthin retinal crystal deposition is a very common finding in patients with prolonged use of the drug. Symptomatic visual loss is less common and correlates with total dosage and possibly patient age. Even with profound visual loss, prognosis for improvement is very good with recognition and discontinuation of the drug.
